# Neurosteroids and Neurotrophic Factors: What Is Their Promise as Biomarkers for Major Depression and PTSD?

**DOI:** 10.3390/ijms22041758

**Published:** 2021-02-10

**Authors:** Felipe Borges Almeida, Helena Maria Tannhauser Barros, Graziano Pinna

**Affiliations:** 1 Graduate Program in Health Science, Federal University of Health Sciences of Porto Alegre (UFCSPA), Rua Sarmento Leite, 245, Porto Alegre 90050-170, Brazil; felipeba@ufcspa.edu.br (F.B.A.); helenbar@ufcspa.edu.br (H.M.T.B.); 2 Department of Psychiatry, The Psychiatric Institute, College of Medicine, University of Illinois at Chicago, 1601 W. Taylor Str., Chicago, IL 60612, USA

**Keywords:** allopregnanolone, BDNF, PTSD, MDD, rodent models, biomarkers

## Abstract

Even though major depressive disorder (MDD) and post-traumatic stress disorder (PTSD) are among the most prevalent and incapacitating mental illnesses in the world, their diagnosis still relies solely on the characterization of subjective symptoms (many of which are shared by multiple disorders) self-reported by patients. Thus, the need for objective measures that aid in the detection of and differentiation between psychiatric disorders becomes urgent. In this paper, we explore the potential of neurosteroids and neurotrophic proteins as biomarkers for MDD and PTSD. Circulating levels of the GABAergic neuroactive steroid, allopregnanolone, are diminished in MDD and PTSD patients, which corroborates the finding of depleted neurosteroid levels observed in animal models of these disorders. The neurotrophic protein, brain-derived neurotropic factor (BDNF), is also reduced in the periphery and in the brain of MDD patients and depressed-like animals that express lower neurosteroid levels. Although the role of BDNF in PTSD psychopathology seems less clear and merits more research, we propose a causal link between allopregnanolone levels and BDNF expression that could function as a *biomarker axis* for the diagnosis of both MDD and PTSD.

## 1. Introduction

Major depressive disorder (MDD) and post-traumatic stress disorder (PTSD) are among the most prevalent, debilitating, and incapacitating mental illnesses that pose a significant disease burden and loss of adjusted life years [1]. Both disorders are believed to emerge as a result of a maladaptive response to stressful events in individuals who fail to develop resilience. MDD and PTSD have similar etiology, manifest overlapping symptoms, and MDD is often seen as a worsening condition in PTSD, which can be accompanied by suicide. The shared symptomatology is relevant because the clinical diagnosis of MDD and PTSD is still based on subjective rather than objective measures that are entirely based on symptom evaluation, following criteria defined by the Diagnostic and Statistical Manual of Mental Disorders (DSM)-V. Even though the DSM-V offers a structured set of symptoms for each disorder, the over reliance on subjective self-reports of symptoms contributes to diagnoses that are misleading at times, mainly in the first medical visits, and in cases characterized by a mild to moderate depression and distress [2]. For this reason, the addition of an array of objective measures of neurobiological parameters would warrant a significant boost in the diagnosis accuracy of psychiatric disorders, including PTSD and MDD. Great progress has been achieved in the study of neurochemical deficits that underlie the manifestation of psychiatric disorders, and several novel biomarker candidates to help in the diagnosis of PTSD and MDD have been proposed.

In this review, we focus on two promising biomarker candidates that may offer a valid biosignature for a better diagnosis and predict occurrence of psychiatric disorders: neurosteroid biosynthesis, such as allopregnanolone and GABAergic congeners, and expression of neurotrophic proteins, including brain-derived neurotrophic factor (BDNF). 

## 2. Biomarkers for Psychiatric Disorders: An Unmet Need

The term “biomarker” refers to a broad category of measurable indicators of the existence or the severity of a disease/disorder, being objective indications of medical state observed from the patient, which can be measured in an accurate and reproducible manner. Thus, biomarkers objectively indicate the pathological state of a subject that correlate to assessed medical symptoms, which are limited to indications perceived by the patient on their mental health disorders. The Biomarkers Definitions Working Group provided an international definition of biomarker for the field of pharmacological clinical trials: “a characteristic that is objectively measured and evaluated as an indicator of normal biological processes, pathogenic processes, or pharmacologic responses to a therapeutic intervention” [3]. 

The development of reliable biomarkers for psychiatric disorders depends on a large understanding of the pathological processes that underlie the specific neuropathology under examination. This can be particularly challenging for psychiatric disorders because of their broad symptomatic characterization and the lack of a sufficiently detailed understanding of the functional abnormalities, either neural or somatic, associated with psychiatric disorders. Nevertheless, significant progress in this field has been made in the last decades, and the development of reliable biomarkers for MDD and PTSD is becoming a realistic possibility. 

There are several kinds of biomarkers that have been proposed for the diagnosis and prognosis of MDD and PTSD, including genetic and epigenetic markers, proteins, and neurohormones [4]. Each of these parameters attempts to reflect part of a theoretical axis of biological alterations that gives origin to symptoms or to the targeted disorders. The origin of such alterations is believed to be largely dependent on the exposure to stressful events, and a complex combination of factors that include the nature of the stressor and the individual’s construct may result in one disorder instead of another. Exposure to stress in a chronic, repeated fashion is believed to be an important risk factor for the development of MDD, while exposure to acute, yet intense, traumatic events, often in individuals that experience chronic stress conditions, may precipitate the development of PTSD. Given that protracted and acute stress play an important role in the etiology of these disorders, the body’s physiological regulation of the stress response becomes an important point of study to contextualize biochemical alterations in MDD and PTSD.

## 3. Neurosteroids

**Figure 1:**
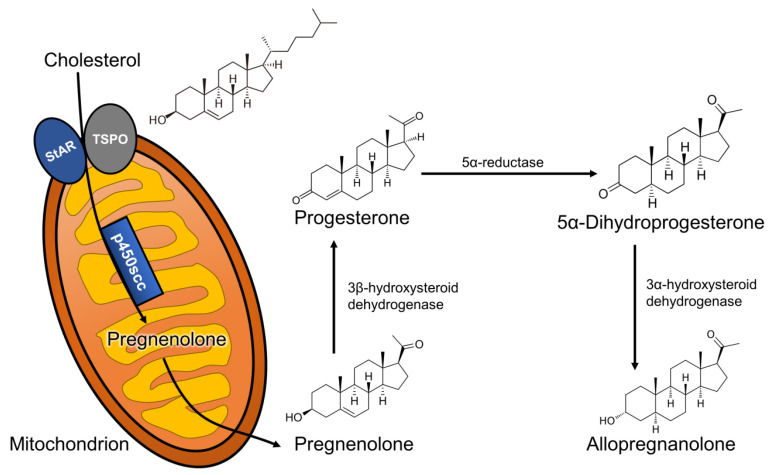
Allopregnanolone biosynthetic pathway starting from cholesterol metabolism. Abbreviations: StAR: steroidogenic acute regulatory protein; TSPO: 18 kDa translocator protein; scc: side-chain cleavage.

Neurosteroids are endogenous steroids synthesized in the central and peripheral nervous system from cholesterol [5] and belong to the broader category of neuroactive steroids, which also include peripherally or artificially synthesized steroids with activity in the brain [6]. The synthesis of neurosteroids, or neurosteroidogenesis, follows a few distinct steps: (1) cholesterol is trafficked to the outer mitochondrial membrane by action of the steroidogenic acute regulatory (StAR) protein; (2) cholesterol is then transported into the inner mitochondrial membrane by the 18 kDa translocator protein (TSPO); (3) its side-chain is cleaved by the CYP11A1 enzyme, which produces the neurosteroid precursor, pregnenolone. Pregnenolone can then be converted to progesterone, which originates a wide array of steroid-derived molecules that include sex and stress hormones, as well as endogenous neuroactive steroids [7]. In this review, we will focus on the neurosteroid pathway (represented in Figure 1) that begins with the reduction of progesterone by the action of the 5α-reductase type I (5α-RI) enzyme, into 5α-dihydroprogesterone, which is subsequently converted by the 3α-hydroxysteroid dehydrogenase (3α-HSD) type III enzyme to 3α,5α-tetrahydroprogesterone (commonly known as allopregnanolone).

Neurosteroids rapidly modulate neuronal excitability due to their affinity to ligand-gated ion channels and other receptors expressed in the synaptosomal membranes of brain cells and neurons. Arguably, the most important neuronal action exerted by neurosteroids is related to the modulation of the inhibitory activity mediated by the neurotransmitter GABA through the allosteric potentiation of the GABA_A_ receptor [8,9,10]. Allopregnanolone and its stereoisomer, pregnanolone, are among the most potent neurosteroids in positively and allosterically modulating GABA_A_ receptors. These GABAergic steroids are found diminished in the cerebrospinal fluid (CSF) [11], serum [12], and plasma [13] of depressed individuals. The expression of the 5α-RI enzyme, which is essential for allopregnanolone synthesis, is reduced in the postmortem prefrontal cortex (Brodman area 9) of depressed individuals [14]. Exogenous administration of allopregnanolone and other GABAergic synthetic neurosteroid analogs resulted in antidepressant and anxiolytic effects, which is believed to be partly due to the role neurosteroids play in the modulation of GABAergic inhibition, thereby also reducing depressive symptoms [15]. The reduced allopregnanolone in the CSF and plasma of depressed individuals [11,12,13] can be increased after antidepressant treatment with selective serotonin reuptake inhibitors (SSRIs), including fluoxetine and fluvoxamine [12].

Affective disorders, including MDD and PTSD, show a strong sex-bias being more prevalent in women than in men, which suggests that sex steroids may play a role. Both progesterone and its metabolite, allopregnanolone, have been the focus of several investigations looking at sex-specific roles of neurosteroids in depressive disorders. Lower serum allopregnanolone levels have also been observed in women suffering from postpartum depression (PPD) [16]. Significant fluctuations in progesterone and allopregnanolone levels during pregnancy, which rise considerably before abruptly decreasing following delivery, may be responsible for the development of PPD pathophysiology [17,18]. From a predictive perspective, the second trimester of pregnancy seems to play an important factor and should be considered in the development of PPD symptoms during the last days of pregnancy and in the post-partum period [19,20].

In female patients with PTSD, CSF, serum, and plasma allopregnanolone levels are also lower than in healthy age-matched controls, which has been associated with enzymatic dysfunction at the levels of 3α-HSD [21,22]. Additionally, women with PTSD show a lower capacity for allopregnanolone synthesis from its precursor, which was confirmed in serum samples [23,24]. Similarly, in males with PTSD, the concentrations of allopregnanolone in the CSF were found lower than in healthy controls [25]. Ratios with allopregnanolone precursors supported a 5α-RI expression/function abnormality. In these patients, the allopregnanolone level decrease was inversely correlated with increased PTSD and depression symptoms. These findings align with previous results that observed a 5α-RI expression downregulation in the human post-mortem brain (Brodmann area 9) of male depressed subjects. Intriguingly, the enzymatic deficits in the allopregnanolone pathway may underlay a biosignature relevant for psychopathology related to dysfunction in reproductive steroid biosynthesis. Allopregnanolone levels were also found to be reduced in the medial orbital frontal cortex of individuals with PTSD in comparison to controls, using postmortem brain tissue samples [26]. A recent study showed that PTSD symptoms were inversely correlated with combined CSF levels of allopregnanolone and pregnanolone, which was not observed in matched trauma-exposed controls [25]. These results are believed to correlate with decreased brain allopregnanolone levels, which are observed in the limbic system areas that are relevant to affect regulation in several animal models of depression and of PTSD (for an in-depth review on this topic, see [27,28,29]). Even though MDD and PTSD share some neurobiological aspects, it is timely to investigate if allopregnanolone downregulation follows a distinct trajectory between the two disorders. Further, it is conceivable that allopregnanolone and pregnanolone levels are compared to the levels of their precursors, calculating ratios that estimate their synthesis rate and also investigating the function and/or expression of the biosynthetic enzymes.

In humans, CSF measurements of neurosteroids are expected to reflect brain levels. However, a study conducted in male and female rats showed a poor correlation of CSF allopregnanolone levels with its content in relevant brain areas, such as the hippocampus and cerebral cortex [30]. Plasma levels were surprisingly predictive of cerebral cortex levels [30], encouraging the extrapolation of peripheral neurosteroid findings to the CNS. Another study in humans found a significantly more robust correlation between CSF and serum free allopregnanolone levels [31]. In human studies in males, however, correlation of steroid levels in the CSF and plasma were either weak or very weak [32]. On the other hand, regardless of the precise correlation within subjects, plasma allopregnanolone levels have been shown to reflect the directional changes predicted in the brain. In addition to plasma, the non-invasive sampling of saliva could offer an alternative to blood draws for the quantification of neurosteroids in that it would reduce the stress associated with blood collection that could affect circulating neuroactive steroid levels, and thereby confounding interpretation of the experimental results. To our knowledge, there have not yet been studies comparing allopregnanolone levels in saliva with blood or CSF levels, but the reliable use of cortisol measurement in saliva [33], suggests that this may be an appropriate direction to focus in future research. Similarly, salivary progesterone levels successfully reflect plasma levels, as confirmed also in studies conducted during pregnancy [34].

Altogether these summaries suggest that more studies should be conducted to establish clear correlation among central neurosteroid biosynthesis changes with those occurring in the periphery, including blood and saliva. These investigations will be crucial in assessing a putative biomarker role of neurosteroids in psychiatric disorders. 

## 4. Neurotrophic Proteins

Exposure to chronic stress can alter BDNF expression, which has also been found abnormal in psychiatric disorders, such as MDD and PTSD. Anxiety and depressive behaviors in humans have been linked with social stress that, when modeled in animals, is associated with changes both in HPA axis function and a down-regulation of BDNF protein and mRNA expression in the hippocampus [35].

**Figure 2:**
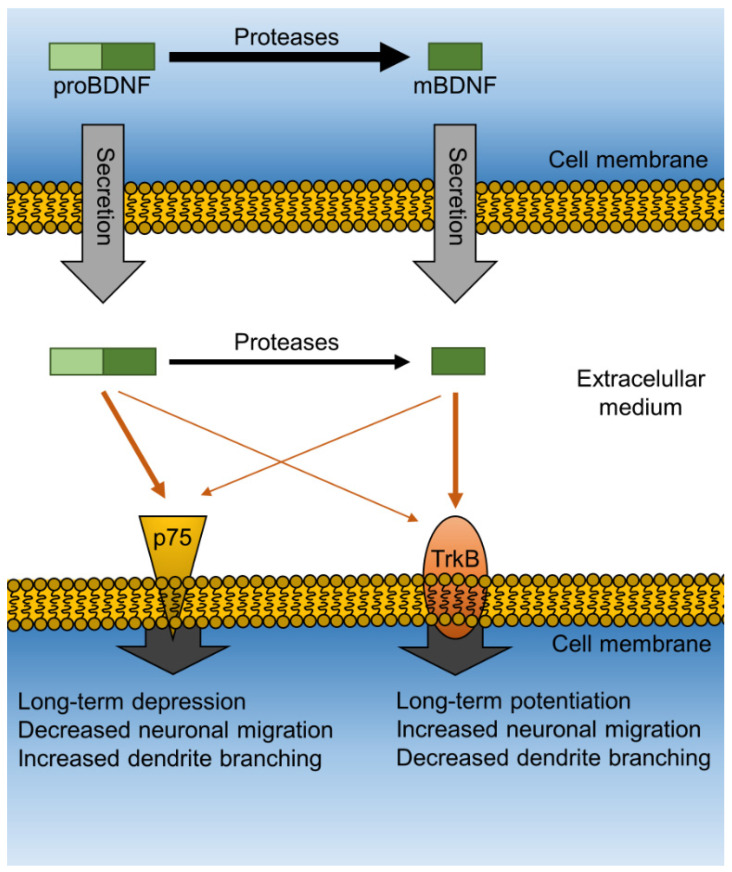
Diagram of the functional role of BDNF isoforms. Conversion of the precursor proBDNF to the mature form mBDNF occurs mainly intracellularly. Both isoforms are secreted and exert their actions on neurotrophin receptor p75 (p75^NTR^) or the tropomyosin receptor kinase B (TrkB). Downstream effects of proBDNF and mBDNF are functionally opposite.

MDD and PTSD are associated with poor ability to maintain and generate functional neurons in key brain regions and circuitry, particularly in the hippocampus, prefrontal cortical regions, and nuclei of the amygdala. Consistently, impaired neurogenesis has been observed in animal models of several psychiatric disorders [36,37]. Neurogenesis and other neurotrophic events, such as maintenance and dendritic spine proliferation and maturation, are generally mediated by neurotrophic proteins, which include the nerve growth factor (NGF), neurotrophin-3 (NT-3), neurotrophin-4 (NT-4), as well as other mediators represented by the steroid dehydroepiandrosterone (DHEA) and its sulfate ester (DHEA-S), among others. However, one of the most important mediators of neurogenesis is undoubtedly BDNF, a neurotrophic protein synthesized by neurons and abundantly expressed in the limbic system, where it plays a role in mood regulation [38]. Like other neurotrophins, BDNF is found in a precursor (proBDNF) and mature (mBDNF) form, and each isoform binds with high affinity to distinct receptors in the brain. While proBDNF binds to the neurotrophin receptor p75 (p75^NTR^), mBDNF exerts its action mainly through activation of the tropomyosin receptor kinase B (TrkB). Although there is some cross-activation of p75^NTR^ by mBDNF and of TrkB by proBDNF that can play a role in some physiological processes, the affinity of this cross-activation remains low and generally does not result in significant overall functional effects. Importantly, the activation of each receptor results in distinct downstream effects that are functionally opposite in nature (Figure 2). Thus, while activation of TrkB leads to neurite outgrowth, stimulates cell migration, increases branching of the dendritic tree, and induces long-term potentiation (LTP), activation of p75NTR reduces neurite length, suppresses cell migration, diminishes branching of the dendritic tree, and induces long-term depression (LTD). Both LTP and LTD are processes that increase or decrease parameters related to synaptic plasticity, respectively [39,40].

There are naturally occurring variations in the BDNF gene that significantly alter its function. Perhaps the most relevant of such variations is a single nucleotide polymorphism (SNP) that occurs in the pro-coding region of the *bdnf* gene (rs6265) called Val66Met (or V66M). Even though this variation does not occur in the mBDNF peptide sequence, its presence leads to inadequate folding and loss of function of the mature neurotrophin. Being highly prevalent (30–50% of carriers in the U.S. and some European countries), this SNP likely plays an important role in BDNF function when related to the development of several neurodegenerative and psychiatric disorders, including MDD [41]. A meta-analysis demonstrated that the V66M polymorphism modulated the relationship between early-life stress and the development of MDD [42]. The role of V66M in PTSD is less clear and less investigated, given that no correlation was found between Val66Met or serum BDNF with the development of PTSD in road traffic accident survivors [43]. On the other hand, a more recent study in U.S. military veterans showed that V66M carriers (i.e., heterozygous for the SNP) exposed to trauma experienced PTSD symptoms with higher severity compared to non-carriers [44].

Like allopregnanolone content, BDNF levels are decreased in depressed patients and respond to antidepressant treatments. For instance, leukocyte BDNF mRNA expression was found to be reduced in depressed patients and restored by antidepressant treatment with escitalopram [45]. Accordingly, peripheral BDNF appears to be reduced in acute depressive episodes and returns to normal during remission [46]. However, a lack of correlation between serum BDNF and severity of MDD symptoms was also reported [47]. In the same direction, animal models of depression such as the chronic unpredictable/mild stress have shown that vulnerable animals that develop depression-like behaviors after exposure to the stressor show a significant reduction in hippocampal BDNF expression, as opposed to resilient animals [48]. Moreover, BDNF overexpression or its knockdown in the hippocampus mediates resilience to chronic stress as measured by behavioral outcomes, granting further support to its central role in the development of MDD symptoms [49].

In PTSD, however, clinical evidence shows changes in BDNF levels are less clear and often contradictory. Some studies suggest that subjects who developed PTSD after exposure to traumatic events present lower blood BDNF levels when compared to those who did not develop the disorder [50] or to healthy controls [51,52]. Another study showed an increase in BDNF levels after antidepressant treatment in PTSD patients, but their baseline levels did not differ from non-PTSD trauma survivors [53]. In another study, however, BDNF tended to be higher in PTSD patients and reached statistical significance when measured in the early stages after trauma [54]. Thus, the clinical data point to a rather complex role of peripheral BDNF levels in PTSD, which are likely dependent on the nature of the trauma and the time after exposure to the traumatic event.

Some preclinical studies have modeled PTSD in rodents and measured brain BDNF to elucidate its central role in this disorder. In the social isolation paradigm, hippocampal BDNF levels have been shown to be decreased in male (but not female) rats [36,55,56]. Because the social isolation model might mimic many of the overlapping characteristics of PTSD and depression [29], it is important to consider expanding this investigation to other stress-based PTSD rodent models in order to provide further support to these findings. Indeed, more recent studies that have examined this topic have found that rats exposed to single prolonged stress (SPS) also present reduced hippocampal BDNF levels [57,58]. These results in animals point to a pivotal, central role of BDNF underlying behavioral dysfunction related to PTSD neurobiology, although the correlation with peripheral BDNF levels is less clear and merits further investigation.

In a complementary strategy to study the role of BDNF in the development of MDD or PTSD after exposure to chronic stress, weaning in an enriched environment has also been studied to verify whether BDNF could drive resilience against the development of these disorders. Post-weaning environmental enrichment has been shown to increase BDNF in the hippocampus when compared to rats reared in standard or isolated conditions [59]. Furthermore, exposure to a socially enriched environment was able to restore the reduction in hippocampal BDNF induced by post-weaning social isolation [60] or by maternal separation [61]. Pre-weaning enrichment was also able to rescue animals from anxiety-like behaviors in adulthood induced by post-weaning maternal separation stress, while increasing BDNF content in the basolateral amygdala [62]. Thus, these findings grant further support for the role of BDNF in driving resilience and contrasting the development of MDD or PTSD, highlighting its potential as a biomarker for the characterization of these disorders.

## 5. The Allopregnanolone and BDNF Link

In preclinical in vivo studies in rodents, a correlation between hippocampal BDNF and allopregnanolone levels is supported by studies that aimed to model depression using a variety of protocols (for review see [27]). In vitro data showing that allopregnanolone promotes proliferation of neural progenitor cells [63], cell survival [64], and neuronal differentiation [65] demonstrate allopregnanolone’s capacity in inducing neurotrophic modifications, thus supporting the hypothesis of a causal—rather than simply correlational—link between these two agents [55]. Many in vivo studies put forth the hypothesis that allopregnanolone can regulate the expression of BDNF in the brain. In male mice, social isolation reduced both allopregnanolone and BDNF in the hippocampus, and long-term treatment with allopregnanolone or SSRIs, administered at low, neurosteroidogenic doses, restored endogenous allopregnanolone and BDNF expression to normal levels quantified in control group-housed rodents [55]. These data were later confirmed by studies conducted in socially isolated male rats administered with allopregnanolone [36]. In female rats, knocking out the pregnane xenobiotic receptor (important for steroidogenesis) reduced hippocampal allopregnanolone concomitantly with a decrease in BDNF expression in the same brain area examined [66]. Chronic treatment with allopregnanolone restored impaired BDNF levels found in the spinal cord of a mouse model of motoneuron degeneration [67]. Low to moderate concentrations of allopregnanolone increased BDNF expression in vitro in response to neuronal damage induced by 6-hydroxydopamine (6-OHDA) administration [68]. 

The precise mechanism, by which allopregnanolone may have induced BDNF expression upregulation, is still elusive, but a recent investigation proposed that allopregnanolone binding at GABA_A_R leads to voltage-gated L-type Ca^2+^ channel opening and subsequent phosphorylation of the calcium/calmodulin-dependent protein kinase II δ3 subunit (CaMKIIδ3), which then increases BDNF mRNA expression [69]. Further studies should determine potential transcription factors/regions for the BDNF gene that allopregnanolone could regulate directly or indirectly.

**Figure 3:**
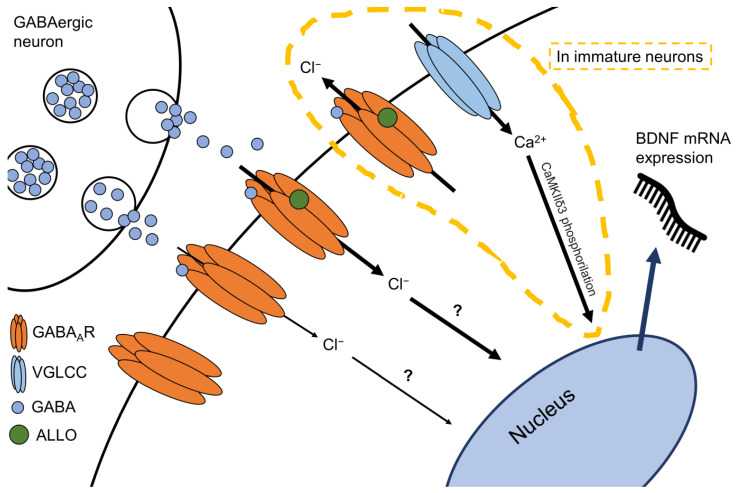
Schematic representation of a putative functional link between allopregnanolone and BDNF. Allopregnanolone (ALLO) potentiation of GABAergic neurotransmission is thought to be involved in BDNF mRNA expression upregulation, though the precise mechanism still needs to be elucidated. One proposed pathway, in immature neurons, is the phosphorylation of the calcium/calmodulin-dependent protein kinase II δ3 subunit (CaMKIIδ3) as a response to allopregnanolone-induced voltage-gated L-type Ca^2+^ channel (VGLCC), which upregulates BDNF gene expression [68].

Altogether, the finding that allopregnanolone biosynthesis and regulation of BDNF expression could be part of the same mechanism (Figure 3) potentially offers a biomarker profile at the interface of the neurosteroid system and neurotrophic factor expression and function suitable to be proposed in the development of precise diagnosis and treatment for MDD and PTSD. Allopregnanolone was recently approved by the US FDA for the treatment of PPD in an intravenous formulation named brexanolone and marketed as Zulresso™ [70]. Its pharmacological action was fast, required only a short course administration, and appeared to be long-lasting. These groundbreaking findings have recently posed allopregnanolone/brexanolone among only two recently US FDA-approved fast-acting antidepressants. The hypothesis of whether allopregnanolone, which is found decreased in several neuropsychiatric disorders, could entail predictions as biomarker of psychiatric disorders deserves further investigation and characterization in preclinical and clinical studies. The angle of looking at a biomarker profiling including allopregnanolone biosynthesis in relation with expression of BDNF is fascinating but remains a topic to be further clarified in future investigations.

## 6. Conclusions

Notwithstanding recent progress in the field, neuropsychiatry still remains a field lacking development of reliable biomarkers to objectively help in the diagnosis and treatment of several debilitating conditions that affect the lives of millions of patients worldwide. Recent investigations in rodent stress models of PTSD and MDD suggest that neurosteroid biosynthesis and expression of BDNF are linked with allopregnanolone regulating the expression of neurotrophic factors [27,36,55]. Given the role of both these neurobiological factors in psychiatric disorders, including MDD and PTSD, the hypothesis that they may serve in biomarker profiling of these conditions is encouraging, and their role in developing individual-tailored treatment to improve response to antidepressants will constitute an enormous advance in the field of neuropsychopharmacology.

## References

[1] Rehm J., Shield K.D. (2019). Global Burden of Disease and the Impact of Mental and Addictive Disorders. Curr. Psychiatry Rep..

[2] Aspesi D., Pinna G. (2018). Could a Blood Test for PTSD and Depression Be on the Horizon?. Expert Rev. Proteom..

[3] Atkinson A.J., Colburn W.A., DeGruttola V.G., DeMets D.L., Downing G.J., Hoth D.F., Oates J.A., Peck C.C., Schooley R.T., Biomarkers Definitions Working Group (2001). Biomarkers and Surrogate Endpoints: Preferred Definitions and Conceptual Framework. Clin. Pharmacol. Ther..

[4] Huang T.-L., Lin C.-C., Makowski G.S. (2015). Chapter Seven—Advances in Biomarkers of Major Depressive Disorder. Advances in Clinical Chemistry.

[5] Baulieu E.E., Robel P., Schumacher M. (2001). Neurosteroids: Beginning of the story. International Review of Neurobiology.

[6] Paul S.M., Purdy R.H. (1992). Neuroactive Steroids. FASEB J..

[7] Mellon S.H., Griffin L.D., Compagnone N.A. (2001). Biosynthesis and Action of Neurosteroids. Brain Res. Rev..

[8] Paul S.M., Pinna G., Guidotti A. (2020). Allopregnanolone: From Molecular Pathophysiology to Therapeutics. A Historical Perspective. Neurobiol. Stress.

[9] Puia G., Vicini S., Seeburg P.H., Costa E. (1991). Influence of Recombinant Gamma-Aminobutyric Acid-A Receptor Subunit Composition on the Action of Allosteric Modulators of Gamma-Aminobutyric Acid-Gated Cl- Currents. Mol. Pharmacol..

[10] Reddy D.S. (2010). Neurosteroids. Progress in Brain Research.

[11] Uzunova V., Sheline Y., Davis J.M., Rasmusson A., Uzunov D.P., Costa E., Guidotti A. (1998). Increase in the Cerebrospinal Fluid Content of Neurosteroids in Patients with Unipolar Major Depression Who Are Receiving Fluoxetine or Fluvoxamine. Proc. Natl. Acad. Sci. USA.

[12] Romeo E., Ströhle A., Spalletta G., di Michele F., Hermann B., Holsboer F., Pasini A., Rupprecht R. (1998). Effects of Antidepressant Treatment on Neuroactive Steroids in Major Depression. Am. J. Psychiatry.

[13] Schüle C., Romeo E., Uzunov D.P., Eser D., di Michele F., Baghai T.C., Pasini A., Schwarz M., Kempter H., Rupprecht R. (2006). Influence of Mirtazapine on Plasma Concentrations of Neuroactive Steroids in Major Depression and on 3α-Hydroxysteroid Dehydrogenase Activity. Mol. Psychiatry.

[14] Agis-Balboa R.C., Guidotti A., Pinna G. (2014). 5α-Reductase Type I Expression Is Downregulated in the Prefrontal Cortex/Brodmann’s Area 9 (BA9) of Depressed Patients. Psychopharmacology.

[15] Maguire J. (2019). Neuroactive Steroids and GABAergic Involvement in the Neuroendocrine Dysfunction Associated with Major Depressive Disorder and Postpartum Depression. Front. Cell. Neurosci..

[16] Nappi R.E., Petraglia F., Luisi S., Polatti F., Farina C., Genazzani A.R. (2001). Serum Allopregnanolone in Women with Postpartum “Blues”. Obstet. Gynecol..

[17] Zorumski C.F., Paul S.M., Izumi Y., Covey D.F., Mennerick S. (2013). Neurosteroids, Stress and Depression: Potential Therapeutic Opportunities. Neurosci. Biobehav. Rev..

[18] Zorumski C.F., Paul S.M., Covey D.F., Mennerick S. (2019). Neurosteroids as Novel Antidepressants and Anxiolytics: GABA-A Receptors and Beyond. Neurobiol. Stress.

[19] McEvoy K., Payne J.L., Osborne L.M. (2018). Neuroactive Steroids and Perinatal Depression: A Review of Recent Literature. Curr. Psychiatry Rep..

[20] Osborne L.M., Gispen F., Sanyal A., Yenokyan G., Meilman S., Payne J.L. (2017). Lower Allopregnanolone during Pregnancy Predicts Postpartum Depression: An Exploratory Study. Psychoneuroendocrinology.

[21] Rasmusson A.M., Pinna G., Paliwal P., Weisman D., Gottschalk C., Charney D., Krystal J., Guidotti A. (2006). Decreased Cerebrospinal Fluid Allopregnanolone Levels in Women with Posttraumatic Stress Disorder. Biol. Psychiatry.

[22] Pinna G. (2020). Allopregnanolone, the Neuromodulator Turned Therapeutic Agent: Thank You, Next?. Front. Endocrinol..

[23] Pineles S.L., Nillni Y.I., Pinna G., Irvine J., Webb A., Arditte Hall K.A., Hauger R., Miller M.W., Resick P.A., Orr S.P. (2018). PTSD in Women Is Associated with a Block in Conversion of Progesterone to the GABAergic Neurosteroids Allopregnanolone and Pregnanolone Measured in Plasma. Psychoneuroendocrinology.

[24] Pineles S.L., Nillni Y.I., Pinna G., Webb A., Arditte Hall K.A., Fonda J.R., Irvine J., King M.W., Hauger R.L., Resick P.A. (2020). Associations between PTSD-Related Extinction Retention Deficits in Women and Plasma Steroids That Modulate Brain GABAA and NMDA Receptor Activity. Neurobiol. Stress.

[25] Rasmusson A.M., King M.W., Valovski I., Gregor K., Scioli-Salter E., Pineles S.L., Hamouda M., Nillni Y.I., Anderson G.M., Pinna G. (2019). Relationships between Cerebrospinal Fluid GABAergic Neurosteroid Levels and Symptom Severity in Men with PTSD. Psychoneuroendocrinology.

[26] Cruz D.A., Glantz L.A., McGaughey K.D., Parke G., Shampine L.J., Kilts J.D., Naylor J.C., Marx C.E., Williamson D.E. (2019). Neurosteroid Levels in the Orbital Frontal Cortex of Subjects with PTSD and Controls: A Preliminary Report. Chronic Stress.

[27] Almeida F.B., Nin M.S., Barros H.M.T. (2020). The Role of Allopregnanolone in Depressive-like Behaviors: Focus on Neurotrophic Proteins. Neurobiol. Stress.

[28] Locci A., Pinna G. (2017). Neurosteroid Biosynthesis Down-Regulation and Changes in GABA _A_ Receptor Subunit Composition: A *Biomarker Axis* in Stress-Induced Cognitive and Emotional Impairment: Neurosteroids and GABA: Biomarkers for Emotions. Br. J. Pharmacol..

[29] Pinna G. (2019). Animal Models of PTSD: The Socially Isolated Mouse and the Biomarker Role of Allopregnanolone. Front. Behav. Neurosci..

[30] Caruso D., Pesaresi M., Abbiati F., Calabrese D., Giatti S., Garcia-Segura L.M., Melcangi R.C. (2013). Comparison of Plasma and Cerebrospinal Fluid Levels of Neuroactive Steroids with Their Brain, Spinal Cord and Peripheral Nerve Levels in Male and Female Rats. Psychoneuroendocrinology.

[31] Kancheva R., Hill M., Novák Z., Chrastina J., Velíková M., Kancheva L., Říha I., Stárka L. (2010). Peripheral Neuroactive Steroids May Be as Good as the Steroids in the Cerebrospinal Fluid for the Diagnostics of CNS Disturbances. J. Steroid Biochem. Mol. Biol..

[32] Martin J., Plank E., Jungwirth B., Hapfelmeier A., Podtschaske A., Kagerbauer S.M. (2019). Weak Correlations between Serum and Cerebrospinal Fluid Levels of Estradiol, Progesterone and Testosterone in Males. BMC Neurosci..

[33] Vining R.F., McGinley R.A. (1987). The Measurement of Hormones in Saliva: Possibilities and Pitfalls. J. Steroid Biochem..

[34] Meulenberg P.M., Hofman J.A. (1989). Salivary Progesterone Excellently Reflects Free and Total Progesterone in Plasma during Pregnancy. Clin. Chem..

[35] Zaletel I., Filipović D., Puškaš N. (2017). Hippocampal BDNF in Physiological Conditions and Social Isolation. Rev. Neurosci..

[36] Evans J., Sun Y., McGregor A., Connor B. (2012). Allopregnanolone Regulates Neurogenesis and Depressive/Anxiety-like Behaviour in a Social Isolation Rodent Model of Chronic Stress. Neuropharmacology.

[37] Zhang X.-Y., Wei W., Zhang Y.-Z., Fu Q., Mi W.-D., Zhang L.-M., Li Y.-F. (2018). The 18 KDa Translocator Protein (TSPO) Overexpression in Hippocampal Dentate Gyrus Elicits Anxiolytic-Like Effects in a Mouse Model of Post-Traumatic Stress Disorder. Front. Pharmacol..

[38] Huang E.J., Reichardt L.F. (2001). Neurotrophins: Roles in Neuronal Development and Function. Annu. Rev. Neurosci..

[39] Borodinova A.A., Salozhin S.V. (2017). Differences in the Biological Functions of BDNF and ProBDNF in the Central Nervous System. Neurosci. Behav. Physiol..

[40] Mizui T., Ishikawa Y., Kumanogoh H., Kojima M. (2016). Neurobiological Actions by Three Distinct Subtypes of Brain-Derived Neurotrophic Factor: Multi-Ligand Model of Growth Factor Signaling. Pharmacol. Res..

[41] Shen T., You Y., Joseph C., Mirzaei M., Klistorner A., Graham S.L., Gupta V. (2018). BDNF Polymorphism: A Review of Its Diagnostic and Clinical Relevance in Neurodegenerative Disorders. Aging Dis..

[42] Zhao M., Chen L., Yang J., Han D., Fang D., Qiu X., Yang X., Qiao Z., Ma J., Wang L. (2018). BDNF Val66Met Polymorphism, Life Stress and Depression: A Meta-Analysis of Gene-Environment Interaction. J. Affect. Disord..

[43] van den Heuvel L., Suliman S., Malan-Müller S., Hemmings S., Seedat S. (2016). Brain-Derived Neurotrophic Factor *Val66met* Polymorphism and Plasma Levels in Road Traffic Accident Survivors. Anxiety Stress Coping.

[44] Pitts B.L., Whealin J.M., Harpaz-Rotem I., Duman R.S., Krystal J.H., Southwick S.M., Pietrzak R.H. (2019). BDNF Val66Met Polymorphism and Posttraumatic Stress Symptoms in U.S. Military Veterans: Protective Effect of Physical Exercise. Psychoneuroendocrinology.

[45] Cattaneo A., Bocchio-Chiavetto L., Zanardini R., Milanesi E., Placentino A., Gennarelli M. (2010). Reduced Peripheral Brain-Derived Neurotrophic Factor MRNA Levels Are Normalized by Antidepressant Treatment. Int. J. Neuropsychopharm..

[46] Fernandes B.S., Berk M., Turck C.W., Steiner J., Gonçalves C.-A. (2014). Decreased Peripheral Brain-Derived Neurotrophic Factor Levels Are a Biomarker of Disease Activity in Major Psychiatric Disorders: A Comparative Meta-Analysis. Mol. Psychiatry.

[47] Molendijk M.L., Spinhoven P., Polak M., Bus B.A.A., Penninx B.W.J.H., Elzinga B.M. (2014). Serum BDNF Concentrations as Peripheral Manifestations of Depression: Evidence from a Systematic Review and Meta-Analyses on 179 Associations (N = 9484). Mol. Psychiatry.

[48] Tornese P., Sala N., Bonini D., Bonifacino T., La Via L., Milanese M., Treccani G., Seguini M., Ieraci A., Mingardi J. (2019). Chronic Mild Stress Induces Anhedonic Behavior and Changes in Glutamate Release, BDNF Trafficking and Dendrite Morphology Only in Stress Vulnerable Rats. The Rapid Restorative Action of Ketamine. Neurobiol. Stress.

[49] Taliaz D., Loya A., Gersner R., Haramati S., Chen A., Zangen A. (2011). Resilience to Chronic Stress Is Mediated by Hippocampal Brain-Derived Neurotrophic Factor. J. Neurosci..

[50] Angelucci F., Ricci V., Gelfo F., Martinotti G., Brunetti M., Sepede G., Signorelli M., Aguglia E., Pettorruso M., Vellante F. (2014). BDNF Serum Levels in Subjects Developing or Not Post-Traumatic Stress Disorder after Trauma Exposure. Brain Cognit..

[51] Dell’Osso L., Carmassi C., Del Debbio A., Dell’Osso M.C., Bianchi C., da Pozzo E., Origlia N., Domenici L., Massimetti G., Marazziti D. (2009). Brain-Derived Neurotrophic Factor Plasma Levels in Patients Suffering from Post-Traumatic Stress Disorder. Prog. Neuro Psychopharmacol. Biol. Psychiatry.

[52] Stratta P., Sanità P., Bonanni R.L., de Cataldo S., Angelucci A., Rossi R., Origlia N., Domenici L., Carmassi C., Piccinni A. (2016). Clinical Correlates of Plasma Brain-Derived Neurotrophic Factor in Post-Traumatic Stress Disorder Spectrum after a Natural Disaster. Psychiatry Res..

[53] Tural Ü., Aker A.T., Önder E., Sodan H.T., Ünver H., Akansel G. (2018). Neurotrophic Factors and Hippocampal Activity in PTSD. PLoS ONE.

[54] Hauck S., Kapczinski F., Roesler R., de Moura Silveira É., Magalhães P.V., Kruel L.R.P., Schestatsky S.S., Ceitlin L.H.F. (2010). Serum Brain-Derived Neurotrophic Factor in Patients with Trauma Psychopathology. Prog. Neuro Psychopharmacol. Biol. Psychiatry.

[55] Nin M.S., Martinez L.A., Pibiri F., Nelson M., Pinna G. (2011). Neurosteroids Reduce Social Isolation-Induced Behavioral Deficits: A Proposed Link with Neurosteroid-Mediated Upregulation of BDNF Expression. Front. Endocrinol..

[56] Pisu M.G., Garau A., Boero G., Biggio F., Pibiri V., Dore R., Locci V., Paci E., Porcu P., Serra M. (2016). Sex Differences in the Outcome of Juvenile Social Isolation on HPA Axis Function in Rats. Neuroscience.

[57] Lee B., Shim I., Lee H., Hahm D.-H. (2018). Effects of Epigallocatechin Gallate on Behavioral and Cognitive Impairments, Hypothalamic–Pituitary–Adrenal Axis Dysfunction, and Alternations in Hippocampal BDNF Expression under Single Prolonged Stress. J. Med. Food.

[58] Shafia S., Vafaei A.A., Samaei S.A., Bandegi A.R., Rafiei A., Valadan R., Hosseini-Khah Z., Mohammadkhani R., Rashidy-Pour A. (2017). Effects of Moderate Treadmill Exercise and Fluoxetine on Behavioural and Cognitive Deficits, Hypothalamic-Pituitary-Adrenal Axis Dysfunction and Alternations in Hippocampal BDNF and MRNA Expression of Apoptosis—Related Proteins in a Rat Model of Post-Traumatic Stress Disorder. Neurobiol. Learn. Mem..

[59] Mosaferi B., Babri S., Mohaddes G., Khamnei S., Mesgari M. (2015). Post-Weaning Environmental Enrichment Improves BDNF Response of Adult Male Rats. Int. J. Dev. Neurosci..

[60] Biggio F., Mostallino M.C., Talani G., Locci V., Mostallino R., Calandra G., Sanna E., Biggio G. (2019). Social Enrichment Reverses the Isolation-Induced Deficits of Neuronal Plasticity in the Hippocampus of Male Rats. Neuropharmacology.

[61] Menezes J., Souto das Neves B.-H., Gonçalves R., Benetti F., Mello-Carpes P.B. (2020). Maternal Deprivation Impairs Memory and Cognitive Flexibility, Effect That Is Avoided by Environmental Enrichment. Behav. Brain Res..

[62] Hegde A., Suresh S., Mitra R. (2020). Early-Life Short-Term Environmental Enrichment Counteracts the Effects of Stress on Anxiety-like Behavior, Brain-Derived Neurotrophic Factor and Nuclear Translocation of Glucocorticoid Receptors in the Basolateral Amygdala. Sci. Rep..

[63] Wang J.M. (2005). The Neurosteroid Allopregnanolone Promotes Proliferation of Rodent and Human Neural Progenitor Cells and Regulates Cell-Cycle Gene and Protein Expression. J. Neurosci..

[64] Djebaili M., Guo Q., Pettus E.H., Hoffman S.W., Stein D.G. (2005). The Neurosteroids Progesterone and Allopregnanolone Reduce Cell Death, Gliosis, and Functional Deficits after Traumatic Brain Injury in Rats. J. Neurotrauma.

[65] Chen S., Wang T., Yao J., Brinton R.D. (2020). Allopregnanolone Promotes Neuronal and Oligodendrocyte Differentiation In Vitro and In Vivo: Therapeutic Implication for Alzheimer’s Disease. Neurotherapeutics.

[66] Frye C.A., Koonce C.J., Walf A.A. (2014). Involvement of Pregnane Xenobiotic Receptor in Mating-Induced Allopregnanolone Formation in the Midbrain and Hippocampus and Brain-Derived Neurotrophic Factor in the Hippocampus among Female Rats. Psychopharmacology.

[67] Meyer M., Garay L.I., Kruse M.S., Lara A., Gargiulo-Monachelli G., Schumacher M., Guennoun R., Coirini H., De Nicola A.F., Gonzalez Deniselle M.C. (2017). Protective Effects of the Neurosteroid Allopregnanolone in a Mouse Model of Spontaneous Motoneuron Degeneration. J. Steroid Biochem. Mol. Biol..

[68] Wang T., Ye X., Bian W., Chen Z., Du J., Li M., Zhou P., Cui H., Ding Y.-Q., Qi S. (2020). Allopregnanolone Modulates GABAAR-Dependent CaMKIIδ3 and BDNF to Protect SH-SY5Y Cells Against 6-OHDA-Induced Damage. Front. Cell. Neurosci..

[69] Chen Z., Wang T., Bian W., Ye X., Li M., Du J., Zhou P., Cui H., Ding Y., Ren Y. (2020). Allopregnanolone Restores the Tyrosine Hydroxylase-positive Neurons and Motor Performance in a 6-OHDA-injected Mouse Model. CNS. Neurosci. Ther..

[70] Meltzer-Brody S., Colquhoun H., Riesenberg R., Epperson C.N., Deligiannidis K.M., Rubinow D.R., Li H., Sankoh A.J., Clemson C., Schacterle A. (2018). Brexanolone Injection in Post-Partum Depression: Two Multicentre, Double-Blind, Randomised, Placebo-Controlled, Phase 3 Trials. Lancet.

